# Raman Spectroscopic Signatures of Hepatic Carcinoma: Progress and Future Prospect

**DOI:** 10.3390/ijms27042023

**Published:** 2026-02-20

**Authors:** Mina Kolahdouzmohammadi, Erfaneh Shaygannia, Kevan Wu, Nicholas Tjandra, Raha Nikoumaram, Nazir P. Kherani, Graziano Oldani

**Affiliations:** 1 Department of Surgery, Faculty of Medicine, University of British Columbia, Vancouver, BC V6T 1Z3, Canada; 2 BC Children’s Hospital Research Institute, Vancouver, BC V5Z 4H4, Canada; 3 Department of Electrical and Computer Engineering, University of Toronto, Toronto, ON M5S 3G4, Canada; 4 Faculty of Pharmaceutical Sciences, University of British Columbia, Vancouver, BC V6T 1Z3, Canada; 5 Faculty of Health Sciences, Simon Fraser University, Burnaby, BC V5A 1S6, Canada; 6 Faculty of Science, University of British Columbia, Vancouver, BC V6T 1Z3, Canada; rnikouma@student.ubc.ca; 7 Department of Material Science & Engineering, University of Toronto, Toronto, ON M5S 3E4, Canada

**Keywords:** Raman spectroscopy, liver cancer, hepatocellular carcinoma, biomarkers

## Abstract

Liver cancer continues to be a predominant cause of cancer-related mortality globally, primarily attributable to late diagnosis and a scarcity of dependable biomarkers for early identification. Raman spectroscopy has emerged as a valuable analytical instrument for liver cancer detection, providing rapid, label-free, and non-destructive molecular profiling of biological specimens. Raman-based methodologies can discern malignant from non-malignant conditions by analyzing small biochemical alterations in biofluids, including blood, urine, and exosomes, as well as in liver tissue, yielding unique spectrum fingerprints. Progress in chemometric analysis, including machine learning models and multivariate statistical methods, has significantly improved the diagnostic precision of Raman spectroscopy, attaining elevated sensitivity and specificity across numerous studies. Furthermore, the integration of complementary techniques, such as surface-enhanced Raman spectroscopy (SERS) and Raman optical activity (ROA) has broadened its prospects for clinical application. This review article elucidates the contemporary applications of Raman spectroscopy in the diagnosis of liver cancer, presents pivotal findings across various sample types, and examines the challenges and future prospects of building Raman-based platforms as dependable diagnostic instruments in oncology.

## 1. Introduction

**Figure 1:**
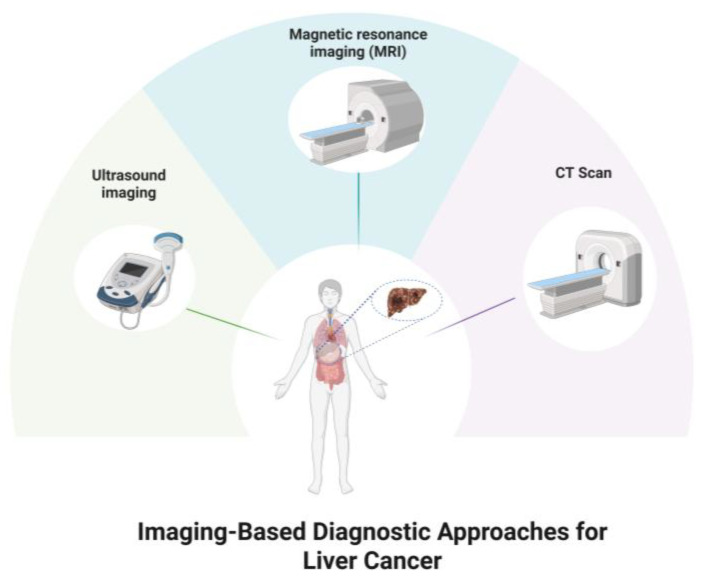
Current Imaging-Based Methods for Liver Cancer Diagnosis (Created in https://BioRender.com).

Liver cancer rates rank sixth among all cancers globally, and is the third major cause of cancer-related mortality [1]. Early-stage liver cancer treatment can ensure a favorable prognosis and comparatively greater survival rate. Late diagnosis, on the other hand, leads to a poor prognosis where the survival rate is relatively low. Absence of nerves in the liver leads to mainly asymptomatic cancer during the early stages [2]. Currently, there are two prevalent screening methods for liver cancer: imaging and serological biomarker assessments [3]. Current imaging modalities comprise ultrasound imaging, magnetic resonance imaging, and computed tomography (Figure 1) [3].

However, these methods possess numerous drawbacks. Sensitivity of ultrasound imaging is highly variable, contingent upon the operator’s expertise and precision of the equipment. Computed tomography and magnetic resonance imaging have low sensitivity for small tumors (less than 1 cm), resulting in a propensity for misdiagnosis or failure to detect [4,5]. Further, an elevated level of the alpha-fetoprotein (AFP) is extensively utilized as a serum biomarker for liver cancer diagnosis [6]. However, even this method suffers from a lack of high sensitivity and specificity for early detection of liver cancer, restricting its clinical utility. In contrast, a punctured biopsy of liver cancer yields not only a definitive pathological diagnosis, significantly aiding in prognosis determination, albeit inducing discomfort to patients. Accordingly, the development of an alternative, complementary, essentially non-invasive, highly sensitive, precise, real-time, and economically viable technique for early detection of liver cancer would be highly beneficial to the field.

**Figure 2:**
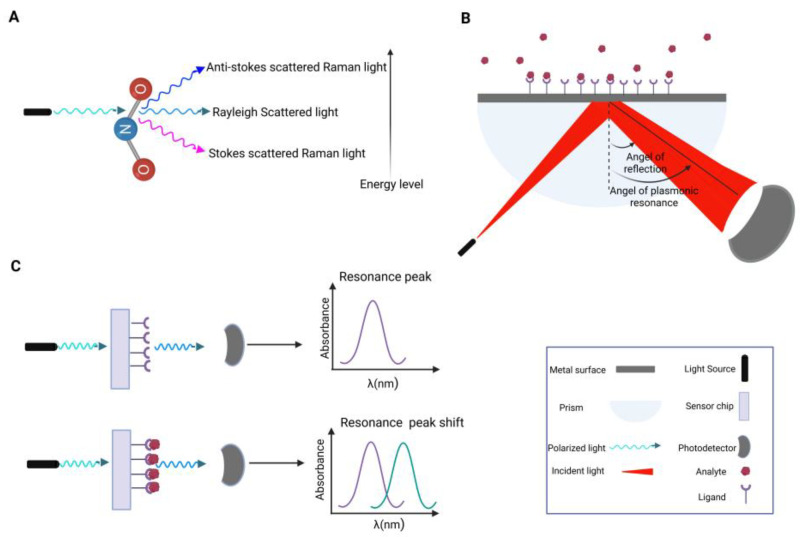
Principles of Raman and SERS-based detection. (**A**) Schematic illustration of Raman scattering: When light interacts with molecular bonds, it has a finite probability of being scattered. The majority of scattered photons undergo elastic scattering—known as Rayleigh scattering—in which the photons retain the same energy, frequency, wavelength, and hence colour, but will in general differ in direction in relation to the incident light. The intensity of Rayleigh scattering typically constitutes about 0.1% to 0.01% of the source radiation. A much smaller proportion of photons, approximately one in ten million, are scattered inelastically, resulting in Raman scattered photons with energies that is usually lower (Stokes scattering) or higher (anti-Stokes scattering). Conversely, in the less probable anti-experimental arrangement (**B**), SPR arises when incident light excites the collective oscillation of free electrons at the interface of a thin metallic film and a dielectric medium. At a specific angle of incidence, at a given wavelength of incident light and refractive index of the surrounding medium, these oscillations propagate parallel to the metal surface. Under these resonant conditions, even minute changes in the refractive index can disrupt the coupling and alter the resonant angle, providing the basis for highly sensitive detection of fine changes in refractive index. This sensitivity makes SPR an invaluable analytical technique for monitoring biomolecular interactions in real time, and thus SPR-based biosensors have been extensively developed for the detection of diverse analytes and clinically relevant biomarkers. (**C**) Laser excitation on a nanostructured sensor chip produces a resonant peak; analyte binding to functionalized ligands shifts the resonance, which is detected by a photodetector as a shift in the peak resonance. SERS amplifies Raman signals through localized surface plasmon resonances (electromagnetic effect) and analyte–metal interactions (chemical effect), enabling highly sensitive molecular detection (Created in https://BioRender.com).

In recent years, surface-enhanced Raman spectroscopy (SERS) has been extensively used in biomedicine, yielding significant outcomes in disease diagnosis and screening [7]. Raman spectroscopy is a sensitive optical technique that probes molecular structure through the inelastic scattering of incident photons by vibrational modes in atoms, molecules or their aggregates, such as crystals, leading to secondary photons—scattered light—which can display changes in phase, polarization, and even energy [8,9] (Figure 2A).

The strength of this method is rooted in the uniqueness of each Raman spectrum for every specific molecule, thus providing a basis for molecular fingerprinting [10]. SERS is a powerful advance over conventional Raman spectroscopy considering its enhancement in inelastic Raman scattering. SERS substrates employ nanostructured metallic surfaces, typically composed of silver or gold, to significantly enhance the light intensity and hence amplify the weak Raman scattering signal from analyte molecules proximal to the surface. Optical illumination of a metal surface or a material with a high density of free charges leads to collective oscillation of surface electrons, a phenomenon known as surface plasmon resonance (SPR) [11]. Under specific conditions, these SPR-induced surface charge oscillations couple with electromagnetic waves, leading to the production of surface plasmon polaritons; the quantum of these oscillations is denoted SPP. The excitation of SPPs represents a fundamental step in SPR-based biosensing [12,13,14] (Figure 2B) where the high intensity of the localized light markedly enhances the surface sensitivity of metallic substrates by increasing the probability of Raman scattering vis-à-vis the target analytes [15,16]. This heightened sensitivity has been widely utilized in virus detection technologies [17,18].

When light interacts with metal nanoparticles instead of a continuous thin film, the resulting plasmonic effect is termed localized surface plasmon resonance (LSPR) [19] (Figure 2C). LSPR induces an intense electric field around the nanoparticle surface [19]. Moreover, when nanoparticles are positioned in close proximity or form aggregates, their coupled plasmonic fields generate a substantially stronger electromagnetic field between particles, leading to the electromagnetic enhancement of Raman scattering from certain chemical species [20,21]. This enhancement occurs through two primary mechanisms: the electromagnetic effect, which is associated with LSPRs in the metallic nanostructures, and the chemical effect, which entails electronic interactions between the analyte and the metal surface [22] (Figure 2B). These combined effects enable SERS to detect even single molecules, providing unparalleled sensitivity and specificity for biomedical and diagnostic applications [23,24,25]. Nowadays, SERS-derived data are combined with machine learning (ML) techniques, which lead to ultra-sensitive detection of molecular species. Thus, the non-invasive characteristics and speed of SERS position it to be an optimal instrument for a manifold of screening applications. In the present context, initial phases, liver cancer, and other malignancies in the incipient stage frequently elicit structural alterations in the associated biomolecules circulating in blood [26]. The variations in the SERS spectra of biofluids can thus signify alterations in the associated tissues and hence enable early detection of illnesses. While Raman and SERS offer promising analytical performance and the potential for cost-effective diagnostics, challenges related to inter-laboratory reproducibility, protocol standardization, and regulatory approval remain important considerations for future clinical translation.

The objective of the present review is to explore potential applications of various Raman spectroscopy techniques in the detection and diagnosis of liver cancer, particularly hepatocellular carcinoma (HCC), while also highlighting avenues for future research and practical implementation. The study selection process adhered to a systematic screening methodology. Initially, 125 research articles were identified using keywords associated with Raman technology, Raman spectroscopy, HCC, intrahepatic cholangiocarcinoma (ICC), and liver cancer. The investigation was performed utilizing PubMed, Google Scholar, and Scopus. Following the screening of titles and abstracts, 85 articles were retained, whereas 40 were excluded for their lack of relevance to the research topic or absence of key terms. The comprehensive evaluation eliminated 32 studies due to absent methodological details, irrelevance to the technique, or unreliable outcomes. This resulted in 53 studies that satisfied the inclusion criteria and were examined in the review. This process guaranteed the inclusion of only studies with adequate methodological and scientific significance.

## 2. Raman Spectroscopy: What Are the Modes and What Are the Applications?

Raman spectroscopy assesses the inelastic scattering of monochromatic light, which produces an ensemble of molecular “fingerprints” of tissues and biofluids where sample preparation requirements are minimal. In biomedicine, Raman spectroscopy and its variants have been investigated for diagnostic and prognostic purposes, considering the ability to detect biochemical changes in lipids, proteins, and nucleic acids, which, combined with multivariate analysis, enables swift categorization [27]. Over time, various improved or modified versions of Raman have been developed to overcome its intrinsically weak signal strength and broaden its application to intricate biological settings. Currently, around 25 distinct types of Raman spectroscopy techniques are used, which include spontaneous Raman, coherent anti-Stokes Raman scattering (CARS) [28], SERS, and tip-enhanced Raman scattering (TERS) [29].

Raman and SERS analyses often necessitate meticulous preprocessing of spectra, including cosmic ray elimination [30], baseline correction [31], and normalization [32], prior to subsequent chemometric or ML evaluation. Multivariate techniques, including principal component analysis (PCA) [33], linear discriminant analysis (LDA) [34], support vector machines (SVM) [35], and deep learning (DL) [36], are currently prevalent, particularly in the analysis of intricate clinical samples. A significant challenge in the application of SERS is the reproducibility of Raman spectra [37], which necessitates the use of standardized substrates and ratiometric methodologies.

Applications of Raman-based technologies in oncology have experienced rapid growth in recent years. Raman spectroscopy has been employed in cancer diagnosis through the analysis of unique chemical compositions [38,39,40]. Several promising pilot studies have shown that Raman spectra can effectively differentiate between malignant and benign skin [41], bladder [42], breast [43], and head and neck [44] tissues with high specificity and sensitivity.

Stimulated Raman histology (SRH), a therapeutic application of stimulated Raman scattering (SRS), generates hematoxylin and eosin-like images of fresh tissue within minutes [45], enabling near-real-time intraoperative identification of brain tumors, and is currently under evaluation for other cancers, including gastrointestinal and urogenital malignancies [46]. Conversely, SERS has become a significant technique for liquid biopsy, as nanoparticles enhance spectral signals from trace biomolecules in serum or plasma samples from patients, and ML classifiers exhibit promising accuracy for early cancer detection [47]. Serum SERS enables the early identification and staging of several malignancies [48], while biomarker-level SERS recognizes proteins, nucleic acids, and cell-surface markers [49], and screening, which together can be extended to personalized and precision medicine.

Consistent with the applications mentioned above, Raman spectroscopy has also been extensively utilized to identify various forms of liver cancer, primarily for the diagnosis of HCC. Here, we explore and summarize the application of Raman spectroscopy and SERS in the diagnosis and treatment of liver cancer vis-à-vis blood serum, liver tissue, blood plasma, and other samples.

## 3. Sample-Based Raman Application in Liver Cancer Treatment/Diagnosis

### 3.1. Blood Serum

The utilization of Raman and SERS methodologies for liver cancer diagnosis has advanced from initial proof-of-concept investigations to highly refined strategies using nanostructures and artificial intelligence (AI), demonstrating consistent enhancement in sensitivity, specificity, and clinical relevance. Most of the contemporary applications of Raman spectroscopy for liver cancer research employ blood serum as the sample (Table 1) [50,51,52,53,54,55,56,57,58,59,60,61,62,63,64,65,66,67,68,69,70,71,72,73,74]. In 2013, the initial application of serum micro-Raman spectroscopy for HCC diagnosis was documented to differentiate sera from cirrhotic patients with and without HCC, achieving approximately 90% accuracy with SVM models but proving ineffective with PCA alone, underscoring the necessity for robust computational methodologies [62]. Soon after, other researchers utilized Ag-colloidal SERS in conjunction with sophisticated classifiers (Partial Least Squares (PLS)-SVM, Artificial Neural Networks (ANN), and orthogonal partial least squares discriminant analysis (OPLS-DA)), resulting in enhanced classification accuracies (>90%) and the identification of metabolic fingerprints, including tryptophan, valine, and nucleic acid peaks associated with HCC [63,64].

Although AFP and AFP-L3 are crucial in HCC diagnoses, numerous SERS investigations have notably improved their detection, showcasing the capacity to enhance traditional tests. Ma et al. (2017) and Ren et al. (2022) demonstrated that functionalized immunochips and antibody-based nanostructures can assess AFP-L3% with high consistency and sensitivity, thereby tackling a persistent clinical problem [60,65]. These focused strategies leverage clinical familiarity and direct translational relevance; nonetheless, they are fundamentally constrained by the moderate sensitivity of AFP in early HCC. Conversely, metabolite- and protein-based profiling [56,64,67] and miRNA-focused assays [55,72] expand the biomarker repertoire, attaining elevated diagnostic accuracies (>95% in certain instances) and providing insights into tumor metabolism and progression beyond AFP alone. Consequently, AFP/AFP-L3 SERS assays serve as a conduit for clinical implementation, whereas multi-omic and AI-enhanced SERS methodologies may delineate the next era of precision diagnostics.

### 3.2. Blood Plasma

Plasma-based spectroscopy offers multiple approaches for liver cancer diagnostics, as summarized in Table 2. Magnetic bead-assisted SERS facilitated multiplex and ultra-sensitive detection of AFP, Carcinoembryonic Antigen (CEA), and Ferritin, achieving pg/mL-level limits of detection and 86.7% accuracy, albeit requiring a multi-step preparation process [76]. Raman spectroscopy and ROA, in conjunction with multivariate statistics, demonstrated modified biomolecular plasma composition, facilitating cancer detection and differential diagnosis of gastrointestinal malignancies; nevertheless, specificity among cancer types was constrained [77]. In obese cirrhotic patients, the amalgamation of infrared (IR), Raman, electronic circular dichroism (ECD), and ROA with sophisticated multivariate models demonstrated robust differentiation of HCC from non-HCC (Area under the receiver operating characteristic (AUROC) 0.961; sensitivity 0.81; specificity 0.857), significantly surpassing individual modalities, although necessitating protracted fluorescence quenching and photobleaching procedures [78]. Benchmarking of IR, Raman, and ROA shows that preprocessing selections significantly influence classification accuracy, offering guidance for reproducibility and underscoring the necessity for uniform data pipelines for clinical application [79].

### 3.3. Liver Tissue

Raman spectroscopy has also been applied to liver tissue samples, with details provided in Table 3 [80,81,82,83,84,85,86,87,88,89]. Lipid signatures detected using Raman imaging demonstrated significant diagnostic accuracy, with Random Forest classification achieving around 86% (sensitivity 76%, specificity 93%) [88]. Optimized pipelines markedly enhanced results: DL models trained on large datasets (>12,000 spectra) attained over 92% accuracy in distinguishing cancer from surrounding tissue and demonstrated consistent effectiveness in differentiating HCC from intrahepatic cholangiocarcinoma (ICC), a clinically challenging task [84]. These clinical investigations frequently surpassed preliminary ex vivo research that depended on limited cohorts and logistic regression, which failed to correctly stratify HCC subclasses. The incorporation of Raman spectroscopy with supplementary methodologies like Matrix-Assisted Laser Desorption/Ionization (MALDI)-Imaging Mass Spectrometry (IMS) enhanced resolution, facilitating both cancer detection and precise grading of HCC, a feat unattainable by Raman alone [81].

Preclinical murine research, conversely, advanced the limits of sensitivity and functional imaging by nanoparticle-based SERS [86]. Gold nanostars and peptide-modified probes achieved signal increases of up to 12-fold relative to unmodified nanostructures, facilitating the detection of microscopic tumors (~250 μm) and early-stage fibrosis that would often elude traditional histology [86]. Fluorescence-guided SERS facilitated the in situ classification and spatial mapping of collagen subtypes, yielding molecular-level fibrosis staging [83]. The most groundbreaking advancement was from the amalgamation of SERS with CT imaging, which not only enhanced sensitivity but also facilitated the swift identification of sub-2 mm lesions within minutes of probe injection, with an accuracy above 91% [80]. Although these findings underscore the significant promise of nanoparticle-assisted SERS for intraoperative navigation and early illness identification, their application is presently constrained by biosafety issues, brief circulation durations, and the absence of extensive human validation.

### 3.4. Other Potential Samples

As summarized in Table 4, blood, urine, and exosomes serve as promising non-invasive sample types for liver cancer detection [90,91,92]. Circulating tumor cells were among the initial targets, with nanoparticle-enhanced SERS tests exhibiting single-cell detection sensitivity (limit of detection: 1 cell/mL) [92]. Despite their technological sophistication, these spectroscopy methods are constrained by the intricacies of nanoprobe manufacturing [92]. Urine-based SERS has evolved as a more accessible option, capturing spectrum signatures of nucleic acids, amino acids, and metabolites, achieving 83–90% sensitivity and specificity for cirrhosis and approximately 85% for HCC, consistently surpassing serum AFP [57]. The low cost, simple processing requirements, and label-free characteristics render urine an appealing biofluid; nevertheless, bigger multicenter trials are necessary to validate its diagnostic reliability.

Exosome profiling has enhanced the liquid biopsy domain by utilizing tumor-derived vesicles as concentrated molecular repositories [90,91]. Nano-gold plasmonic substrates facilitated very reproducible exosomal SERS spectra, achieving diagnostic performance that surpasses AFP, with sensitivities and specificities approaching 95–100% in differentiating HCC from viral hepatitis cohorts [91]. The domain has evolved to sophisticated AI-assisted frameworks, wherein deep learning, coupled with large language models “ChatExosome”, amalgamated spectral and molecular characteristics to attain over 94% accuracy in a substantial patient cohort, notably maintaining robust performance in AFP-negative instances (87.5%) [90]. This signifies a pivotal advancement towards clinically pertinent solutions, tackling both diagnostic sensitivity and interpretability. These liquid biopsy studies illustrate a progressive evolution: from proof-of-concept blood assays to practical urine-based screening, culminating in exosome-focused platforms that integrate nanoscale sensitivity with AI-driven precision, positioning them at the forefront of translational potential.

### 3.5. Raman Application in Liver Cancer Cell Lines

Raman-based methodologies have been extensively utilized in in vitro liver cell models, offering regulated settings to analyze spectrum biomarkers and evaluate methodological advancements prior to application on patient samples. Single-cell investigations employing laser tweezers Raman spectroscopy integrated with deep neural networks successfully distinguished hepatocytes from several liver cancer cell lines, revealing metabolic markers associated with differentiation state, highlighting the promise of optical tweezers for high-resolution, label-free diagnostics [94]. Complementary investigations on freshly uncultured primary cells and mixed tumor/non-tumor populations revealed that AI-assisted Raman spectroscopy can attain classification accuracies nearing 90–93% in pure samples; however, performance diminished with a reduced proportion of tumor cells, highlighting the challenge posed by spectral heterogeneity [95]. SERS nanoprobes have been developed to investigate functional and molecular markers in cultured cells: dual-reporter nanoflower probes facilitated ratiometric quantification of carboxylesterase-1 activity in HepG2 cells with integrated internal normalization, while dual-nanoprobe systems specifically targeted lncRNA DAPK1-215, an oncogenic regulator of migration and invasion, achieving precise intracellular detection with minimal cytotoxicity [96]. In addition to cell culture, spiking experiments in blood confirmed the capability of identifying circulating tumor cells at concentrations as low as 1 cell/mL utilizing TiO_2_@Ag nanoprobes, demonstrating proof-of-principle for liquid biopsy applications [97]. These in vitro models collectively demonstrate the adaptability of Raman and SERS platforms, encompassing metabolic phenotyping, functional enzyme assays, and mutation-specific nucleic acid detection, thereby underscoring their significance as a link between mechanistic cellular investigations and clinically pertinent liquid biopsy diagnostics.

## 4. Conclusions

Raman and SERS have established themselves as versatile and powerful tools in the study and diagnosis of liver cancer, spanning applications from tissue analysis to liquid biopsy and in vitro models. Tissue-based studies have shown that Raman spectroscopy, especially when used with AI classifiers or multimodal platforms, can accurately tell the difference between malignant and non-malignant liver tissue and even grade tumors [95]. These kinds of studies show how useful Raman can be as a supplement to histopathology and as a guide for making decisions during surgery. In addition, liquid biopsy techniques have expanded Raman’s use in non-invasive diagnostics: urine-based SERS [57] has been shown to be better than AFP for finding cirrhosis and HCC, exosome-derived spectra have come close to perfect accuracy [91], and AI-driven exosome platforms [90] now have strong diagnostic power even in patients who are negative for AFP—this is arguably the most clinically important breakthrough so far. Blood-based assays, including CTC detection [92], remain technically impressive but face challenges in scalability and standardization.

The biochemical changes identified by Raman spectroscopy and SERS in HCC can be understood within the larger context of tumor metabolic reprogramming. This metabolic reprogramming is a key characteristic that allows malignant liver cells to continue proliferating, adapt to their tumor microenvironment, and evade immune detection [98]. Core metabolic shifts in HCC include the dysregulation of glucose utilization, lipid biosynthesis, and amino acid metabolism. These shifts not only contribute to the progression of the tumor but also play a role in modulating the immune response. Raman-detectable molecular signatures, such as altered vibrations in lipids, proteins, and nucleic acids, reflect these metabolic changes [99]. These signatures provide label-free insights into the biochemical remodeling associated with cancer, observable across cells, tissues, and bodily fluids. Rather than serving as isolated biomarkers, these spectral features collectively represent the integrated metabolic and microenvironmental changes that occur during hepatocarcinogenesis. This supports the biological relevance of Raman-based diagnostics for characterizing the disease and monitoring therapeutic responses.

In vitro and cell-line studies continue to play an indispensable role, enabling precise exploration of Raman biomarkers, functional enzyme activity, and oncogenic nucleic acid signatures under controlled conditions [96]. These models serve as innovative testbeds, providing mechanistic insight and proof-of-principle evidence prior to translating it for patient-derived samples. However, their diagnostic accuracies, often high in homogeneous settings, must be interpreted cautiously, as they do not fully capture the complexity of clinical biofluids or tumor microenvironments.

Despite the frequently reported high diagnostic accuracy in Raman-based liver cancer studies, such findings must be interpreted cautiously due to methodological limitations. These include small or single-center cohorts, cohort imbalances, reliance on internal validation, and heterogeneous analytical pipelines. Such factors increase the likelihood of overfitting and reduce generalizability across different clinical populations. Particularly for AI- and deep learning-based approaches, it is crucial to assess dataset size, external and multicenter validation, and regulatory feasibility before considering any clinical translation to be reliable. Consequently, the performance metrics currently reported should be viewed as evidence of technical feasibility rather than definitive clinical effectiveness. In addition, cost–benefit justification and operational robustness under real-world conditions remain critical determinants for adoption. Addressing these factors through substrate standardization, harmonized analytical pipelines, external multicenter trials, and regulatory-grade validation frameworks will be essential to enable reliable clinical implementation of Raman-based diagnostics.

Importantly, tissue-based Raman diagnostics and emerging liquid-biopsy platforms appear closest to near-term clinical translation, whereas in vivo nanoparticle-enabled SERS approaches remain largely exploratory due to unresolved challenges related to biosafety, regulatory approval, and long-term biocompatibility [100].

In a nutshell, translating Raman/SERS technology into clinical applications can be a game-changer in diagnosis and precision medicine, recognizing its high sensitivity, real-time sensing, and low cost (>$10/sample) platform. Moreover, as discussed, it does not require complicated sample preparation or invasive methods to collect samples. The amount of sample needed is as low as 2 µL, and using a 785 nm laser typically does not harm biological samples.

## Figures and Tables

**Table 1:** Utilization of blood serum in Raman spectroscopy investigations for liver cancer diagnosis.

Authors/Year	Groups and Sample Size	Sample Preparation	Raman/SERS Technique	Analysis Method	Key Targets	Main Findings	Advantages	Limitations
Taleb et al., 2013 [62]	37 Cirrhosis patient + HCC, 34 Cirrhosis patient	Serum prepared as dried drops and freeze-dried	Micro-Raman spectroscopy, 785 nm laser	SVM, PCA (comparison)	Global serum biochemical profile	SVM classification achieved 84.5–90.2% accuracy for dried serum and 86–91.5% accuracy for freeze-dried serum, PCA alone failed to discriminate cirrhotic patients with HCC and those without HCC	Non-invasive, label-free, rapid diagnostic approach	Proof-of-concept only, further validation and spectral feature details needed
Li et al., 2015 [63]	45 Liver cancer patient (pre-treatment), 42 Liver cancer patient (post-treatment), 45 Liver cirrhosis patient	2 μL serum mixed with 2 μL Ag colloid ultrasonically	SERS, 633 nm laser	SVM, PLS-DA, ANN	Serum metabolite	Classification accuracy: 91.5% (PLS-SVM), 89.2% (PLS-DA), 90.3% (PLS-ANN)	Non-invasive hepatic disease screening with high diagnostic accuracy	Preliminary study, and needs validation in larger, independent cohorts
Xiao et al., 2016 [64]	47 HCC patient, 60 Healthy control	Au@Ag core–shell nanorods (Au@AgNRs) mixed directly with serum	SERS, 785 nm laser	OPLS-DA classification	Serum metabolites (tryptophan, phenylalanine, proline, valine, adenine, thymine), AFP-related spectral peaks	Unique metabolic fingerprints identified, OPLS-DA achieved AUC = 0.991 for HCC vs. controls	Non-invasive, label-free, multiplex metabolite detection, high diagnostic accuracy	Broad metabolite identities, requires specialized SERS substrate
Ma H. et al., 2017 [65]	15 HCC patient; 16 BLD patient; 1 Healthy control	Functionalized immunochips (Ag/MBA/anti-AFP) + immunogold nanoprobes (DSNB–AuNP/anti-AFP-L3), serum pipetted onto chips	SERS, 633 nm laser	frequency shift (MBA) + intensity change (DSNB)	Total AFP, AFP-L3, AFP-L3%	Combined SERS detection of AFP and AFP-L3% enabled early and accurate HCC diagnosis	High reproducibility, simplified AFP-L3% detection vs. conventional assays	Very limited healthy controls
Yang et al., 2017 [66]	6 Liver cancer patient, Healthy control	Au@Ag core–shell nanostructures, serum diluted in PBS and incubated in antibody-coated 96-well plates	SERS, 532 nm laser	Peak intensity measurement of 4-MBA reporter band, correlated with AFP concentration via calibration curve	AFP	Au@Ag nanostructure able to generate stronger SERS signals for AFP detection	Extremely high sensitivity, high specificity, stable core–shell nanoparticles	Small patient cohort, requires multiple immunoassay steps
Ma H. et al., 2018 [67]	21 HCC patient, Normal serum	Self-assembled Ag nanoparticle chip with PTCA linker, serum samples incubated directly on chip	SERS, 633 nm, and 785 nm lasers	HCA	Protein biomarkers in serum	PTCA-based SERS enabled discrimination of protein biomarkers (including early HCC markers) and differentiated structurally similar proteins without requiring antibodies	Label-free, antibody-free discrimination of protein biomarkers with high versatility	Small sample size
Yu et al., 2018 [68]	104 Liver cancer patient, 100 Nasopharyngeal cancer patient, 95 Healthy control	Membrane electrophoresis of serum proteins, cut band, dissolve in acetic acid, mix with AgNPs	SERS, 785 nm laser	Multivariate analysis (PCA vs. PLS) + SVM classifier	Serum protein vibrational signatures	Training set accuracy 95.09%, test set accuracy 90.67%. Sensitivity for liver cancer early stage (T1–T2): 83.3%, advanced (T3–T4): 94.1%. Specificity ~93.68%. PLS-SVM outperformed PCA-LDA and PCA-SVM	Non-invasive serum-based method, simultaneous detection of multiple cancer types in a single test	Testing accuracy (90.67%) lower than training, advanced stages detected more reliably than early stages
Feng et al., 2020 [69]	3 Liver cancer patient, 3 Healthy control	serum antigen (hCE1) bound between (i) 4-MBA labeled AgNP–anti-hCE1 “SERS tags” and (ii) Fe_3_O_4_@SiO_2_@AgNP–anti-hCE1 magnetic substrates	SERS magnetic immunosensor, 638 nm laser	Raman signal quantification at 1609 cm^−1^, linear calibration	Human carboxylesterase 1 (hCE1)	Detection limit of hCE1 as low as 0.1 ng/mL	Ultra-sensitive, non-invasive, reproducible and stable	Requires nanocomposite synthesis, tested on limited human samples
Gao et al., 2020 [70]	58 Liver cancer patient; 30 Breast cancer patient; 60 Healthy control	Hydroxyapatite (HAp) nanoparticles for albumin adsorption–exfoliation: Mix 50 µL serum with HAp at 1:2	SERS, 785 nm laser	PLS + SVM	Serum albumin	100% accuracy (liver cancer vs. normal), 96.68% accuracy (Breast cancer vs. normal)	Label-free, non-invasive, good linear detection range (1–10 g/dL), lower detection limit < clinical hypoalbuminemia threshold (3.5 g/dL)	Focused only on albumin, not other biomarkers
Cheng et al., 2021 [71]	124 HCC patients, 124 Healthy controls	NBC: AgNP-decorated ZnO nanorods on cellulose paper: 6 µL serum directly dropped onto NBC	SERS on 3D nanoplasmonic paper chip, 785 nm laser	Spectrum-based deep learning (CNN) for binary classification, baseline/smoothing/normalization preprocessing, k-fold crystal violet (CV) and external validation	Biomolecules in human serum	External set: 91% accuracy, 90% sensitivity, 92% specificity (50 HCC vs. 50 healthy) Chip performance: intra-chip RSD 7.5–11.2%; inter-batch RSD 3.5%	Antibody-free, low-cost paper chip, POCT-friendly, minimal prep (one drop), robust ratiometric-free but deep-learning-assisted readout, good reproducibility	Purely serological (no mechanistic biomarker quantification)
Wu et al., 2021 [72]	92 clinical sera across various groups (AFP-negative, pre-/post-hepatectomy, recurrence status, BCLC stages); 2 Healthy controls	Fractal AuNP SERS tags + Ag-coated magnetic nanoparticles (AgMNPs), 1% serum used for multiplex SERS assay	Multiplex SERS, 633 nm laser	Quantification of SERS intensity vs. miRNA concentration	miRNA-122, miRNA-223, miRNA-21 biomarkers	AgMNP-based magnetic separation improved SERS activity, achieved strong linear correlation between SERS signal and log (miRNA concentration)	Multiplex capability, ultra-sensitive, works across different HCC disease stages	Assay complexity (dual nanoparticle system, DNA functionalization, magnetic separation)
Gao et al., 2021 [74]	25 Liver cancer patients T1 stage; 23 Liver cancer patient T2–T4 stages; 35 Healthy controls	HAp microspheres used to preferentially adsorb and release serum albumin: 2 mg HAp mixed with 100 μL serum	SERS, 785 nm laser	PCA + LDA	Serum albumin	Diagnostic accuracy: 90% (T1 vs. normal), 96.55% (T2–T4 vs. normal), PCA-LDA distinguished cancer stages effectively	Label-free, non-invasive, sensitive detection, preserves albumin structure during extraction, higher diagnostic accuracy than previous plasma-SERS methods	Advanced stages detected more reliably than early stages
Gurian et al., 2021 [56]	72 HCC patients; 72 Healthy controls	5 µL serum dropped on AgNP-decorated plasmonic paper substrate: spectra collected directly	SERS, 785 nm	PCA-LDA with RDCV	Metabolic fingerprints	Average classification accuracy ≈ 81% with PCA-LDA (≤4 PCs), RDCV confirmed the model relied on bands from uric acid, hypoxanthine, ergothioneine, and glutathione	Fast; low-cost, portable setup, label-free multi-marker readout, rigorous validation via RDCV, interpretable PCs linked to metabolites	Moderate accuracy
Suksuratin. et al., 2022 [75]	30 CCA patients; 30 Healthy controls	The 2.3 μL of serum was dropped onto a sample well made by attaching a flat washer onto a mirror-grade stainless steel plate	Raman spectroscopy, 785 nm laser	PCA-LDA, peak-height LDA, k-fold cross-validation (k = 5)	Biomolecular markers in serum: cholesterol, methionine/tryptophan, amide III, beta-carotene	CCA vs. controls distinguished with 86.7% sensitivity, 96.7% specificity	Rapid, label-free, minimally invasive, cost-effective, high accuracy	Mostly advanced-stage CCA
Li et al., 2022 [58]	17 HCC patients	Transcatheter arterial chemoembolization (TACE) for HCC	SERS, 785 nm laser	Spectral preprocessing, biomarker peak assignment, PLS-based machine learning models (LDA/SVM/KNN), and cross-validation	Circulating nucleic acids, collagen, and amino acid changes before vs. after TACE	Within 3 days post-TACE, significant spectral shifts (nucleic acid, collagen, amino acid peaks) enabled accurate early prediction of therapeutic response	Rapid, minimally invasive	Requires validation in larger cohorts
Gao et al., 2022 [59]	40 Liver cancer patients, 32 Prostate cancer patients, 30 Healthy controls	Serum mixed with AgNPs at 1:1 ratio, 5 µL mixture dropped on aluminum slide	SERS, 785 nm laser	Fluorescence background removed, spectra normalized, PLS for dimension reduction, SVM for cancer classification	Metabolic fingerprints	98.04% diagnostic accuracy and 100% accuracy in the testing set for distinguishing cancer patients from healthy controls	Non-invasive, label-free, minimal sample prep, coffee-ring gives strong, reproducible hot-spots, fast measurement, high diagnostic performance	Aluminum slide + drying step required, potential variability in nanoparticle batches and drying dynamics
Ren et al., 2022 [60]	1 HCC patient, 6 Healthy controls	AFP antigens diluted in PBS/NaCl, incubated on antibody-functionalized SERS substrates, washed and dried before measurement	SERS, 785 nm laser	SERS spectral enhancement compared with ELISA reference	AFP and AFP-L3	EIT-like substrate provided order-of-magnitude SERS signal enhancement, enabled accurate AFP-L3% quantification, results strongly correlated with ELISA	High sensitivity, label-free, improved AFP-L3% detection, early HCC diagnostic potential	Small sample size
Ou et. al., 2024 [61]	35 Liver cancer patients, 64 Healthy controls	Serum diluted 1:2 with deionized water; 2 µL of cleaned Ag@SiO_2_ sol dropped on pre-cleaned silicon wafer, dried before SERS test	SERS, 633 nm, and 785 nm lasers	PCA, PLS-DA, OPLS-DA (+SNV preprocessing)	Serum biomolecules (DNA, amino acids, lipids, carbohydrates)	OPLS-DA+SNV: accuracy, sensitivity, and specificity >97%, PLS-DA risk of overfitting, spectral changes reflect cancer-related metabolism	Non-invasive, rapid, high sensitivity/specificity	Cancer subtype not specified, larger/early-stage validation needed
Huang et al., 2023 [55]	15 HCC patients, 15 Healthy controls	Au NA substrate modified with Cy3-H1 DNA, blocked with MCH, miR-224 solution added, followed by Rox-H2 hybridization before SERS measurement; serum diluted to 1% PBS	SERS	Linear fitting and ROC curves	miR-224 (HCC-associated circulating miRNA), specificity checked vs. miR-21, -16, -199a, -125b, -122	Ultrasensitive detection of miR-224 in serum (LOD ~0.34 fM), distinguishing HCC patients from healthy controls, differentiating BCLC stages, and monitoring patients before/after hepatectomy with high accuracy (AUC = 1)	Dual-mode (cross-checks SERS/FL), tiny sample volume, shelf-stable substrates	Single biomarker (miR-224), specialized nanofiber and optics
Sheng et al., 2024 [54]	Nude mouse HCC model: 4 groups based on tumor progression stage: 0, 10, 20, and 30 days post-tumor implantation (4 mice per group)	Raman reporter (4-MBA/DTNB)–labeled AuNPs conjugated with hairpin DNA were assembled on Fe_3_O_4_@cDNA via EDC/NHS coupling, mixtures introduced into a PDMS microfluidic chip (with magnet-assisted mixing) for serum/target testing	SERS, 785 nm laser	Multivariate spectral analysis, biomarker dynamics profiling	AFP, manganese superoxide dismutase (MnSOD)	Dual biomarker SERS detection in serum achieved ultra-low detection limits, strong reproducibility, and results consistent with ELISA. Enabled real-time monitoring of biomarker changes during tumor progression in mice	Ultra-sensitive, rapid (5 min), pump-free portable microfluidic chip, stable and reproducible, high agreement with ELISA	Validated only in mouse serum, requires further clinical testing
Sun et al., 2024 [53]	60 Liver cancer patients (27: T1–T2, 33: T3–T4), 40 Healthy controls	low abundance proteins (LAPs) isolated by Protein A column (IgG removal) and cold ethanol fractionation (albumin depletion), LAPs mixed with AgNPs at 1:1 ratio, 5 µL mixture dropped on aluminum slide and air-dried for SERS measurement	SERS, 785 nm laser	PCA-LDA algorithm	LAPs associated with liver cancer at different stages	Demonstrated high-precision detection of liver cancer across different stages using label-free SERS targeting low-abundance proteins	Label-free, high precision, stage-specific applicability	Weak at differentiating between cancer stages
Yang et al., 2024 [52]	79 Liver cancer patients, 80 Healthy controls	Serum samples were mixed with AgNPs (prepared by hydroxylamine reduction of AgNO_3_) and centrifuged, concentrated AgNPs used as SERS substrate for signal enhancement	SERS, 532 nm laser	Wavelet Transform and DL	Serum biomolecular spectral features	Accuracy, sensitivity, and specificity >97.0% with Morlet wavelet + EfficientNetV2	Non-invasive, ultra-high accuracy, wavelet transforms preserved multi-scale features, DL overcame nonlinear	Requires computational infrastructure
Ji et al., 2025 [50]	4 HCC patient; 1 Acute myocardial infarction (AMI) patient, 2 Healthy	Clinical serum mixed with aptamer-modified nanofingers	SERS, 785 nm laser	Dynamic Raman mapping & Linear regression for quantification	AFP and Cardiac troponin I (cTnI, AMI biomarker)	AFP in patient serum (21 ng/mL) detected within 3 min, absent in healthy serum, detection sensitivity: 0.01 ng/mL AFP, cTnI in AMI serum (6.796 ng/mL) detected at 1 min, biomarker Raman spectra captured selectively, avoiding interference from other serum molecules	Ultra-rapid (<3 min) detection, high sensitivity and specificity (single-molecule SERS level), no sample pre-treatment required (works directly in serum), quantitative via biomarker/aptamer Raman ratio	Large-scale clinical validation still needed, potential variability in nanofinger fabrication

4-Mercaptobenzoic Acid (MBA), 5,5′-Dithiobis (2-nitrobenzoic acid) (DSNB), Benign liver disease (BLD), Cholangiocarcinoma (CCA), Convolutional Neural Network (CNN), Discriminant Analysis (DA), Hierarchical cluster analysis (HCA), Limit of Detection (LOD), Nano Particle (NP), Nanoplasmonics biosensing chip (NBC), Perylenetetra carboxylic acid (PTCA), Point-of-Care Testing (POCT), Repeated double cross-validation (RDCV), Relative Standard Deviation (RSD), standard normal variable (SNV), Area under the curve (AUC).

**Table 2:** Liver cancer diagnosis using Raman spectroscopy and blood plasma.

Authors/Year	Groups and Sample Size	Sample Preparation	Raman/SERS Technique	Analysis Method	Key Molecular/Cellular Targets	Main Findings (Quantitative/Qualitative)	Advantages	Limitations
Bai et al., 2019 [76]	39 suspected liver cancer patients	Samples incubated with antibody-functionalized magnetic beads, and then the captured antigens were incubated with reporter-encoded AuNP SERS tags for detection	m-SERS (magnetic-induced SERS), 633 nm laser	Calibration curve	AFP, CEA, Ferritin	LOD: AFP 0.15 pg/mL, CEA 20 pg/mL, Ferritin 4 pg/mL; 86.7% accuracy with triple-antigen detection	Multiplex, ultra-sensitive, rapid, portable	Multi-step preparation
Králová et al., 2024 [77]	29 HCC patients (stages A–C), 27 CC (colorectal carcinoma) patients, 57 Pancreatic cancer patients, 78 Healthy controls	Plasma cleaned (centrifuge + 0.45 µm filter), NaI quench + 12 h photobleach	Raman spectroscopy, 532 nm laser + ROA	PCA-(band-based LDA)	Altered biomolecular composition of plasma	Raman spectroscopy+ ROA+ multivariate statistics enables both cancer detection and differential diagnosis of gastrointestinal cancers	Non-invasive, disease-specific discrimination	Lack of specificity between cancer types
Hribek et al., 2024 [78]	20 HCC patient, 17 Healthy controls	Prior to Raman, 10 mg NaI/100 µL plasma was added and samples were photobleached (280 mW, 12 h) to suppress fluorescence before spectral acquisition	spontaneous Raman, 532 nm laser	Whole-spectrum and feature-selected PLS-DA; mean-centering/autoscaling; baseline correction (BubbleFill) and SNV; Savitzky–Golay smoothing	strong carotenoid bands	In obese cirrhotic patients, combining IR, Raman, ECD, and ROA spectroscopies with multivariate analysis discriminated HCC from non-HCC with AUROC 0.961 (sens. 0.81, spec. 0.857), outperforming single-method models	Rapid, label-free, small-volume plasma, multi-modal spectra capturing concentration and conformation, strong combined-model discrimination	Needs specialized instruments, lengthy Raman/ROA workflows (fluorescence quench + photobleach, 24 h acquisition) may limit immediate clinical deployment
Vrtělka et al., 2025 [79]	68 Cirrhosis+ HCC; 91 Cirrhotic controls without HCC	Blood plasma samples were analyzed using IR spectroscopy, Raman spectroscopy, and ROA	Raman spectroscopy, 532 nm laser, ROA, IR spectroscopy	ML classifiers (PLS-DA, SVM, Random Forest)	Plasma-derived spectral signatures of HCC vs. cirrhosis	Pre-processing choice strongly affects accuracy	Comprehensive benchmarking of spectral preprocessing in liquid biopsy, provides guidelines for reproducibility	More standardized approach needed for data processing to improve reliability and clinical applications

**Table 3:** Utilization of liver tissue in Raman spectroscopic investigation for liver cancer diagnosis.

Authors/Year	Groups and Sample Size	Sample Preparation	Technique Raman/SERS	Analysis Method	Key Molecular/Cellular Targets	Main Findings (Quantitative/Qualitative)	Advantages	Limitations
Pence et al., 2015 [89]	5 HCC patients, 5 adenocarcinoma patients, 5 Healthy controls	Samples thawed at room temperature, positioned on a stage, and multiple spectra were acquired from different regions	Raman spectroscopy with InGaAs detector, 1064 nm laser	Sparse multinomial logistic regression (SMLR)	Spectral markers: retinol, heme, biliverdin/quinones, lactic acid, collagen, nucleic acids	Specific Raman bands enabled discrimination between healthy vs. cancerous liver tissue, lower accuracy for HCC subclassification	Minimizes autofluorescence, high sensitivity, real-time diagnostic potential	Small sample size, limited subclassification accuracy
Tolstik et al., 2015 [88]	23 HCC patients	Tissue sections mounted on CaF_2_ slides, and Raman spectra were acquired after pre-bleaching autofluorescence (2 s)	Raman imaging spectroscopy, 785 nm laser	Random Forest classifier	Tissue-level lipid molecular signatures (fatty acids)	Random forest classification accuracy: 86% overall (76% sensitivity, 93% specificity)	Label-free, spatially resolved, leverages tissue lipid biochemistry for classification	Moderate sample size, histological heterogeneity may affect generalizability
Andreou et al., 2016 [86]	3 Myc-driven genetically engineered mouse models of HCC, 1 Ink4A/Arf–/– mouse model of histiocytic sarcoma, 2 Healthy controls	Gold NPs synthesized, silica-coated with Raman dye (BPE/IR-780), purified, dispersed in buffer, and injected (150 µL; 22 nM gen (GENERATION)-1 or 3 nM gen-2) 12–18 h before imaging	SERS nanoparticles, 785 nm laser	Raman mapping with Direct Classical Least Squares (DCLS) model, biodistribution quantified by Raman intensity and gold content	Nanoparticle uptake in liver Kupffer cells vs. tumor tissue (reduced uptake in tumors)	SERS NPs accumulated ~40-fold higher in healthy liver tissue vs. HCC tumor tissue, tumor margins precisely delineated by Raman imaging, microscopic tumors (~250 μm) detected by SERS, SERS NPs stable under laser illumination, no photobleaching observed	High tumor delineation accuracy, detection of microscopic lesions, high photostability compared to ICG, single injection provides long imaging window	Requires specialized Raman imaging systems (not yet widely available clinically), nanoparticle regulatory approval more complex than small molecules
Biscaglia et al., 2019 [87]	C57BL/6J mice transgenic for human SerpinB3 (TG-SB3)	A 30 mg frozen liver sample was homogenized in RIPA lysis buffer with ceramic beads, and the resulting lysate was used for SERRS measurements	SERRS imaging/spectroscopy, Near infra-red (NIR) laser	Pearson correlation to reporter spectrum (threshold > 0.6)	SB3 on liver cancer cells via HBV PreS1 peptide	No cytotoxicity; mouse liver shows SERRS up to ~3–4 h, gone by 6 h	High specificity (PEG spacer), bright/stable SERRS, low toxicity, peptide stable, simple readout	No clinical patient testing
Poojari et al., 2021 [85]	6 cohorts, 3 specimens/cohort: (1) saline, (2) Cet-PLGA-b-PEG NP, (3) CA4 + 2ME, (4) PLGA-b-PEG-CA4 NP + PLGA-b-PEG-2ME NP, (5) Cet-PLGA-b-PEG-CA4 NP + Cet-PLGA-b-PEG-2ME NP, (N) healthy liver	Snap-frozen liver and tumor tissues thawed and mounted on CaF_2_ slides for Raman spectroscopy	Confocal Raman spectroscopy, 532 nm laser	PCA, LDA	EGFR, microtubules, lipids, amide-I	Raman spectroscopy discriminated HCC vs. healthy liver and treatment groups	High sensitivity, label-free, rapid	Preclinical (mouse) ex vivo only, small per-cohort specimens
Xiang et al., 2021 [82]	120 ICR mice: 60 Fibrosis model, 60 Healthy (~30 mice per subgroup)	Gold nanostars (GNSs) or GA-PEG-SH-modified GNSs (GLTTs) injected via tail vein; liver tissues sliced into 100 μm sections with tissue slicer, slices mounted on silicon wafers for SERS detection	SERS, 785 nm laser	Savitzky–Golay smoothing + fluorescence background subtraction	Carbohydrate (glucose/glycogen), lipids, proteins, amino acids in liver parenchymal cells	GLTTs produced ~12.85× stronger SERS signals in liver tissue compared to unmodified GNSs	High sensitivity, liver-targeted specificity, reproducibility, PEG improved dispersion and biosafety, non-invasive diagnosis potential	Only S1 fibrosis tested, short-term safety confirmed but long-term biosecurity risks remain
Kirchberger-Tolstik et al., 2021 [81]	36 HCC patients	10 μm tissue sections mounted on CaF_2_ slides for Raman	Raman spectroscopic imaging, 785 nm laser + MALDI-IMS	Multivariate analysis	Proteins, lipids, collagen, glycogen	Raman alone predicted HCC vs. non-cancer with 88% sensitivity, 80% specificity, 84% accuracy. MALDI- IMS differentiated HCC grades (well vs. moderate/poor) with 100% sensitivity and 80% specificity	Label-free, non-destructive molecular differentiation of HCC and tumor grade	Small sample size, Raman alone less effective for grading
Huang et al., 2023 [84]	98 HCC patients, 22 ICC patients	5 µm frozen sections prepared on microtome and fixed to slides; minimal pre-treatment before Raman	Raman spectroscopy, 785 nm laser	CNN trained on 12,000 spectra (50 per tissue, 500–2000 cm^−1^ range) Compared with PLS-DA, Random Forest, XGBoost Preprocessing: baseline subtraction, Savitzky–Golay smoothing, cosmic ray removal Raman imaging: SMCR (self-modelling curve resolution) + HCA clustering	Carotenoids, aromatic AAs, amide I, lipids, nucleic acids, saccharides	Cancer vs. adjacent: Acc 92.6%, Sens 90.8%, Spec 94.6%. HCC vs. ICC: Acc 82.4%. Stage: 78.3%. Differentiation: 72.3%. AUCs 0.783–0.965. Raman images delineate boundaries; 3D subcellular protein/lipid maps	Label-free, minimal preparation, high-accuracy tissue diagnosis, margin mapping (2D/3D), intraoperative feasibility, AI handles heterogeneity	Device-to-device spectral differences, spontaneous Raman is weak (speed/quality trade-off), needs standardization
Jiang et al., 2024 [80]	5 Orthotopic liver cancer (at days 4 and 14) mouse, 5 Healthy controls	Mice injected with AuNPs (200 μL, 50 mg/mL Au); sacrificed at 24 h, liver excised, sliced, and fixed on glass slides, spectra acquired directly from tissue slices	SERS, 633 nm laser	AI-driven spectral analysis (Random Forest classifier, ROC/AUC metrics)	Nucleotides, lipids, proteins (amide bands, β-sheet, phosphate stretches)	CT + SERS achieved 91.38% accuracy in distinguishing healthy vs. HCC liver; Nucleotide-to-lipid ratio identified as a key biomolecular marker for HCC; Early-stage HCC (~2 mm) detectable by CT/SERS within 5 min post-injection	Integrates morphology (CT) + molecular profiling (SERS), early detection capability (2 mm tumors), high diagnostic accuracy, biocompatible AuNPs with prolonged circulation	Small sample size, preclinical mouse-only study

Hepatitis B Virus (HBV), Polyethylene glycol (PEG), Epidermal Growth Factor Receptor (EGFR), Indocyanine Green (ICG).

**Table 4:** Application of Raman spectroscopy for liver cancer diagnosis using a variety of samples.

Authors/Year	Experimental Model	Groups and Sample Size	Sample Preparation	Raman/SERS Technique	Analysis Method	Key Molecular/Cellular Targets	Main Findings (Quantitative/Qualitative)	Advantages	Limitations
Pang et al., 2018 [92]	Human peripheral blood	8 HCC patients, 5 Breast cancer patients, 5 Healthy controls	Samples were incubated with the anti-ASGPR-Fe_3_O_4_@Ag MNPs, and then the isolated cells were incubated with the anti-GPC3-Au@Ag@DTNB for SERS detection	SERS, 785 nm laser	Magnetic enrichment + SERS spectral analysis	Circulating tumor cells (CTCs)	LOD: 1 cell/mL, linear range 1–100 cells/mL	Highly sensitive, dual-marker selectivity	Small sample size, requires nanoprobe synthesis
Dawuti et al., 2022 [57]	Human urine and blood serum	49 Liver Cirrhosis patient; 55 HCC patient, 50 Healthy control	5 µL urine mixed with 5 µL Ag colloid (1:1), mixture dropped on aluminum foil, and spectra collected with Raman micro-spectrometer	SERS, 785 nm laser	SVM	Urinary metabolites (nucleic acids, amino acids)	For liver cirrhosis: sensitivity, specificity, and accuracy 83–90%. For HCC: sensitivity, specificity, accuracy ~85%. SERS outperformed serum AFP for HCC detection	Non-invasive, label-free, rapid, cost-effective, higher sensitivity than AFP	Needs multicenter validation
Elkady et al., 2023 [91]	Human whole blood, serum-isolated exosomes	20 HCV-HCC, 20 hepatitis C virus (HCV) patients, 20 Healthy controls	Whole blood and exosome samples were placed in a cuvette with a nano-gold plasmonic substrate (200 × 200 nm) to enhance Raman signal	SERS, 785 nm laser	Peak discrimination, CLSI EP12-A2	Circulating tumor–derived exosomes	HCC: 95% sensitivity, 100% specificity; HCV: 100% sensitivity, 100% specificity	Non-invasive, label-free, high accuracy vs. AFP, standardized performance reporting	Specialized chip/laser (~1500 nm) needs
Qin et al., 2024 [93]	Human blood plasma and Extracellular Vesicle (EVs)	15 non-cancer liver disease, 10 liver cancer, 10 lung cancer, 10 breast cancer patients,10 Healthy controls	Isolated EVs using B@MOF capture bubbles, incubated with antibody/Raman-reporter functionalized AuAg nanobox SERS nanotags to form B@MOF–EV–SERS complexes for detection	SERS	Multiplex SERS signal profiling of EV biomarkers	EV surface biomarkers: CD63, EGFR, HER2, EpCAM	2 min EV isolation with ~87% capture efficiency and detection limit of 70 EVs/mL, enabled multiplexed single-EV profiling	Rapid, non-invasive, high-efficiency capture, portable, multiplexed detection	Complex assay component, requires broader clinical validation
Yang et al., 2025 [90]	Human plasma -derived Exosomes	125 HCC patient: 61 AFP^+^, 65 AFP^−^; 40 Healthy	Exosome solutions dropped on AuNP-coated SERS substrate arrays (self-assembled AuNP monolayers)	SERS	Feature Fusion Transformer (FFT, patch-based 1D self-attention DL model) + Retrieval-Augmented Generation (RAG) with LLMs	Nucleic acids, lipids, metabolites, exosome marker proteins: CD9, CD63, CD81	An LLM-centered AI (“ChatExosome”) analyzing exosome SERS spectra accurately detects HCC (94.1% in clinical samples) and still performs well in AFP-negative cases (87.5%), enabling interactive, interpretable diagnosis	High accuracy, interpretable, scalable to other cancers	Requires AuNP substrate, computational intensity

Clinical and Laboratory Standards Institute (CLSI), Large language models (LLMs).

## Data Availability

No new data were created or analyzed in this study. Data sharing is not applicable to this article.

## Abbreviations

The following abbreviations are used in this manuscript:

MBA	4-Mercaptobenzoic Acid
DSNB	5,5′-Dithiobis (2-nitrobenzoic acid)
AMI	Acute myocardial infarction
AFP	Alpha-fetoprotein
AI	Artificial intelligence
ANN	Artificial neural network
AUC	Area under the curve
AUROC	Area under the receiver operating characteristic curve
BCLC	Barcelona Clinic Liver Cancer
BLD	Benign liver disease
CEA	Carcinoembryonic antigen
cTnI	Cardiac troponin I
CCA	Cholangiocarcinoma
CTCs	Circulating tumor cells
CLSI	Clinical and Laboratory Standards Institute
CARS	Coherent anti-Stokes Raman scattering
CNN	Convolutional neural network
CV	Crystal violet
DL	Deep learning
DA	Discriminant analysis
DCLS	Direct classical least squares
ECD	Electronic circular dichroism
EGFR	Epidermal growth factor receptor
EV	Extracellular vesicle
GLTTs	GA-PEG-SH-modified GNSs
GNSs	Gold nanostars
HCC	Hepatocellular carcinoma
HCV	Hepatitis C virus
HCA	Hierarchical cluster analysis
hCE1	Human carboxylesterase 1
HAp	Hydroxyapatite
HBV	Hepatitis B virus
IMS	Imaging mass spectrometry
IR	Infrared
ICC	Intrahepatic cholangiocarcinoma
ICG	Indocyanine green
LLMs	Large language models
LOD	Limit of detection
LDA	Linear discriminant analysis
LSPR	Localized surface plasmon resonance
LAPs	Low-abundance proteins
ML	Machine learning
MnSOD	Manganese superoxide dismutase
MALDI	Matrix-assisted laser desorption/ionization
NP	Nanoparticle
NBC	Nanoplasmonics biosensing chip
NIR	Near-infrared
OPLS-DA	Orthogonal partial least squares–discriminant analysis
PLS	Partial least squares
PTCA	Perylenetetracarboxylic acid
POCT	Point-of-care testing
PEG	Polyethylene glycol
PCA	Principal component analysis
ROA	Raman optical activity
RSD	Relative standard deviation
RDCV	Repeated double cross-validation
SMCR	Self-modelling curve resolution
SMLR	Sparse multinomial logistic regression
SNV	Standard normal variate
SERS	Surface-enhanced Raman spectroscopy
SPR	Surface plasmon resonance
SPPs	Surface plasmon polaritons
SRS	Stimulated Raman scattering
SRH	Stimulated Raman histology
SVM	Support vector machine
TERS	Tip-enhanced Raman scattering
TG-SB3	Transgenic for human SerpinB3
